# Evaluation of Real-World Studies on Management of Relapsed Multiple Myeloma After BCMA-Directed Therapy Failure from U.S. Academic Centers and USMIRC

**DOI:** 10.3390/curroncol33060355

**Published:** 2026-06-12

**Authors:** Nagham Youssef, Maha Hameed, Shebli Atrash, Barry Paul, Abdullah Mohammad Khan, Hira Shaikh, Christopher Strouse, Andrew Vegel, Zena Chahine, Anita Mazloom, Muhammad Salman Faisal, Taha Al-Juhaishi, Omar Alkharabsheh, Anas Zayad, Carmel Awadallah, Jordan Snyder, Zahra Mahmoudjafari, Muhammad Umair Mushtaq, Jeries Kort, Alma Habib, Forat Lutfi, Al-Ola Abdallah, Mansi Shah, Prerna Mewawalla, Sarah Waheed

**Affiliations:** 1 Levine Cancer Center, Atrium Health, Wake Forest University School of Medicine, Charlotte, NC 28204, USA; naghamyoussef4567@gmail.com (N.Y.); shebli.atrash@advocatehealth.org (S.A.); barry.paul@advocatehealth.org (B.P.); 2 Internal Medicine Department, Florida State University, Sarasota, FL 34236, USA; maha.hameed@med.fsu.edu; 3 Division of Hematology, The Ohio State University, Columbus, OH 43210, USA; abdullah.khan@osumc.edu; 4 Division of Hematology, Oncology, and Blood & Marrow Transplantation, University of Iowa, Iowa City, IA 52242, USA; hira-shaikh@uiowa.edu (H.S.); christopher-strouse@uiowa.edu (C.S.); andrew-vegel@uiowa.edu (A.V.); 5 Division of Bone Marrow Transplant, University of Kentucky, Lexington, KY 40536, USA; zena.chahine@uky.edu; 6 Division of Hematology and Oncology, Mitchell Cancer Institute, University of South Alabama, Mobile, AL 36604, USA; amazloom@health.southalabama.edu; 7 Division of Bone Marrow Transplant, University of Oklahoma Health Sciences, Oklahoma City, OK 73104, USA; muhammadsalman.faisal@ouhealth.com (M.S.F.); taha-aljuhaishi@ou.edu (T.A.-J.); 8 Division of Hematology and Oncology, University of Cincinnati, Cincinnati, OH 45267, USA; alkharor@ucmail.uc.edu; 9 Department of Internal Medicine, Hamad Medical Corporation, Doha P.O. Box 3050, Qatar; anaszayad46@gmail.com; 10 Division of Internal Medicine, St John’s Episcopal Hospital, Far Rockaway, NY 11691, USA; awadallah@qaffaf.com; 11 Division of Hematologic Malignancies & Cellular Therapeutics, University of Kansas Medical Center, Westwood, KS 66205, USA; jsnyder1@kumc.edu (J.S.); zmahmoudjafari@kumc.edu (Z.M.); mmushtaq@kumc.edu (M.U.M.); jkort@kumc.edu (J.K.); ahabib3@kumc.edu (A.H.); flutfi@kumc.edu (F.L.); aabdallah@kumc.edu (A.-O.A.); 12 Division of Hematology, Rutgers Cancer Institute of New Jersey, New Brunswick, NJ 08903, USA; shahmr@rutgers.edu; 13 Division of Hematology and Cellular Therapy, Allegheny Health Network Cancer Institute, Pittsburgh, PA 15224, USA; prerna.mewawalla@ahn.org; 14 Division of Hematology and Bone Marrow Transplant, Johns Hopkins Hospital, Baltimore, MD 21231, USA

**Keywords:** multiple myeloma, BCMA-directed therapy, CAR-T cell therapy, bispecific antibodies, teclistamab, talquetamab, treatment sequencing, antigen switching, real-world outcomes, relapsed/refractory multiple myeloma (RRMM)

## Abstract

B-cell maturation antigen (BCMA)-directed therapies (BDTs) have transformed relapsed/refractory multiple myeloma treatment, but optimal post-failure sequencing remains undefined. We evaluated real-world outcomes from three retrospective, multicenter analyses. Study 1 compared BCMA CAR-T and BCMA T-cell engagers (TCEs) in BDT-exposed patients (*n* = 95). Study 2 evaluated teclistamab in BDT-exposed versus BDT-naïve patients (*n* = 164). Study 3 examined talquetamab (GPRC5D-targeting TCE) in heavily pretreated patients (*n* = 68). CAR-T therapy achieved superior outcomes versus TCE (overall response rate [ORR] 79% vs. 51%, *p* < 0.001; median overall survival [OS] 30 vs. 12 months, *p* = 0.008). Teclistamab-treated BDT-exposed patients had lower ORR (53% vs. 68%, *p* = 0.02) and shorter median progression-free survival (PFS; 2.5 vs. 9.7 months, *p* = 0.01) compared with BDT-naïve patients. Administration < 6 months post-BDT showed inferior outcomes (hazard ratio [HR] 2.5 for PFS; HR 2.9 for OS). Talquetamab achieved an ORR of 68.3% among BDT-exposed patients, with significantly lower response rates when administered < 6 months post-BDT or when BDT was the immediate preceding treatment (56.8% vs. 84.6% and 48% vs. 80.6%, respectively). Treatment-free intervals of ≥6 months between T-cell-redirecting therapies improved efficacy and survival. Post-BDT sequencing should prioritize CAR-T therapy when feasible, allow >6-month intervals before BDT re-challenge, and utilize non-BCMA targets for early relapse or BDT-refractory disease.

## Simple Summary

Treatments that target B-cell maturation antigen (BCMA) have dramatically improved outcomes for patients with relapsed or refractory multiple myeloma, but the best strategy after these therapies stop working remains unclear. This study analyzed real-world data from three multicenter cohorts to examine how patients respond to different treatments after BCMA-directed therapy. The results showed that BCMA CAR-T therapy produced deeper responses and longer survival than BCMA T-cell engagers, while patients receiving teclistamab after prior BCMA therapy experienced reduced effectiveness, especially when treatments were given within six months of each other. In contrast, talquetamab, which targets a different antigen (GPRC5D), remained active in many patients, supporting the value of switching targets after BCMA failure. These findings suggest that treatment timing and antigen selection are critical and may guide future clinical trials, treatment guidelines, and real-world strategies to improve long-term outcomes for patients with multiple myeloma.

## 1. Introduction

Multiple myeloma (MM) is an incurable plasma cell malignancy characterized by cycles of remission and relapse. Patients require repeated therapeutic interventions over the course of the disease [1,2]. Recently, the introduction of B-cell maturation antigen (BCMA)-targeted therapies (BDTs) has revolutionized treatment options, offering hope for patients with heavily pretreated or refractory disease [3,4,5,6]. BCMA, a transmembrane receptor expressed almost exclusively on plasma cells, is an ideal target for immunotherapy because of its restricted expression and role in plasma cell survival [7,8,9].

Six BCMA-directed therapies (BDTs) have received approval from the U.S. Food and Drug Administration (FDA) for the treatment of relapsed/refractory multiple myeloma (RRMM). These include three bispecific T-cell engagers (teclistamab, elranatamab, and linvoseltamab), two chimeric antigen receptor (CAR) T-cell therapies (idecabtagene vicleucel [ide-cel] and ciltacabtagene autoleucel [cilta-cel]), and one antibody–drug conjugate (belantamab mafodotin) [3,4,10,11,12,13,14,15]. Patients with triple-class refractory disease, defined by resistance to a proteasome inhibitor, immunomodulatory agent, and anti-CD38 monoclonal antibody, have demonstrated robust response rates to these agents [16].

As BDTs are increasingly used earlier in the disease course, relapse after prior BDT exposure has become a significant therapeutic challenge. Resistance mechanisms, including antigen modulation, T-cell exhaustion, and immune dysfunction, underscore the need to define effective sequencing strategies, whether through retargeting BCMA or switching to alternative antigens, such as G protein-coupled receptor, family C, group 5, member D (GPRC5D) or Fc receptor-homolog 5 (FcRH5) [17].

In this study, we integrated data from three retrospective, multicenter, real-world analyses conducted across US academic centers in collaboration with the U.S. Myeloma Innovations Research Collaborative (USMIRC). We evaluated therapeutic outcomes and sequencing strategies after BDT failure, compared BCMA-targeted CAR-T and bispecific TCEs, and assessed the efficacy of teclistamab and talquetamab in patients with RRMM. Through comparative analysis of these three datasets, our goal is to provide insights into the relative efficacy, optimal timing, and sequencing of post-BDT for RRMM, thereby guiding future treatment strategies and prospective trial designs.

## 2. Materials and Methods

We synthesized results from three independent, retrospective, multicenter studies conducted within the USMIRC network between January 2022 and May 2024. Because each study had distinct eligibility criteria and analytic approaches, cross-study comparisons are descriptive and hypothesis-generating rather than formal statistical comparisons. Study 1 (Snyder-1) compared BCMA-directed CAR-T therapy (ide-cel or cilta-cel) and BCMA bispecific TCEs (teclistamab or elranatamab) in previously BDT-exposed patients (*n* = 95) [18]. Study 2 (Snyder-2) compared outcomes with teclistamab in BDT-exposed (*n* = 60) and BDT-naïve patients (*n* = 104) [19]. Study 3 (Vegel) assessed the safety and efficacy of talquetamab in heavily pretreated myeloma (*n* = 68), including 63 patients with prior BDT [20].

Eligible patients were adults (≥18 years) with RRMM who were treated at USMIRC centers. Study-specific inclusion and exclusion criteria have been previously published [18,19,20]. Patients were followed from treatment initiation until death, loss to follow-up, or data cutoff. Standard definitions were applied for BDT exposure and refractoriness, high-risk cytogenetics, triple-refractory disease, treatment-free interval (≤6 vs. >6 months), and extramedullary disease.

The primary endpoint was overall response rate (ORR) per International Myeloma Working Group (IMWG) criteria [21]. Secondary endpoints included ≥very good partial response (VGPR), complete response, progression-free survival (PFS), and overall survival (OS). Time-to-event outcomes were estimated by the Kaplan–Meier method and compared with the log-rank test, with hazard ratios (HRs) derived from Cox proportional hazards models. Descriptive statistics and appropriate comparative tests were used for baseline characteristics. All tests were two-sided, with *p* < 0.05 considered significant; given the exploratory retrospective design, *p*-values are descriptive and not adjusted for multiplicity. Analyses were performed with R software version 4.4.2 [22].

Descriptive statistics were used across all studies to summarize patient demographics, disease characteristics, and efficacy outcomes. In Study 1, Fisher’s exact test and the Wilcoxon rank-sum test were used to analyze categorical and continuous variables, respectively, including ORR. In Study 2, PFS and OS were estimated with the Kaplan–Meier method. In Study 3, categorical and continuous variables were compared with χ^2^ or Fisher’s exact tests and analysis of variance, respectively, with OS, PFS, and duration of response estimated by Kaplan–Meier methods; Cox regression used to evaluate associations with baseline characteristics.

All studies were approved by institutional review boards at participating centers, with waiver of informed consent due to the retrospective design. Data were de-identified prior to analysis.

## 3. Results

### 3.1. Snyder-1: BCMA CAR-T Versus BCMA TCEs After Prior BDT Exposure [18]

Of the 95 patients, 42 (44%) received BCMA CAR-T (ide-cel, *n* = 32; cilta-cel, *n* = 10) and 53 (56%) received BCMA TCE (teclistamab, *n* = 52; elranatamab, *n* = 1). Baseline characteristics were comparable between groups, including median age (66 years [range, 42–83]), high-risk cytogenetics (43% vs. 57%; defined according IMWG criteria), and extramedullary disease (43% vs. 42%).

The ORR was higher in the CAR-T cohort than in the TCE group (79% vs. 51%; *p* < 0.001), with deeper responses (≥VGPR: 64% vs. 47%; *p* < 0.001). Median PFS favored CAR-T therapy (6 months [95% CI, 5–14] vs. 2 months [95% CI, 1–8]; *p* = 0.057), while OS was significantly longer (30 months vs. 12 months; *p* = 0.008). The predominant prior BDT was antibody–drug conjugate therapy in the CAR-T cohort (64%) and CAR-T in the TCE cohort (75%). Non-relapse mortality was similar between the groups (8% vs. 15%; *p* > 0.05). Progressive disease was the leading cause of death in both cohorts (33% vs. 29%). A comparison of baseline characteristics and efficacy outcomes for BCMA-directed CAR-T therapy and BCMA TCEs after prior BDT exposure in Snyder-1 is shown in Table 1.

### 3.2. Snyder-2: Teclistamab Outcomes in BDT-Exposed Versus Naïve Patients [19]

Out of 164 patients who received teclistamab between January 2022 and May 2024, 60 (37%) had had prior BDT exposure. Median age was 66 (range: 37–84 years) in the BDT-exposed cohort versus 69 years (range: 46–87 years, *p* = 0.092) in the BDT-naïve cohort. There were similar rates of Revised International Staging System (R-ISS) stage III disease (28% vs. 28%) in BDT-exposed versus BDT-naïve cohort, respectively. The most common prior BDT class was CAR-T (68%) followed by ADC (37%); 5 patients (8.3%) had prior TCE. Compared with BDT-naïve patients, BDT-exposed individuals had a higher median number of prior lines of therapy (7 vs. 4; *p* < 0.001), more frequent triple-refractory disease (88% vs. 73%; *p* = 0.022), and a higher rate of prior autologous stem cell transplantation (75% vs. 59%; *p* = 0.035). The BDT-exposed group exhibited lower ORR (53% vs. 68%; *p* = 0.02), shorter PFS (2.5 months [95% CI, 1.4–8.9] vs. 9.7 months [95% CI, 4.5–NR]; *p* = 0.01), and inferior OS (9.1 months vs. not reached; *p* = 0.08), though the latter difference was not statistically significant.

**Figure 1:**
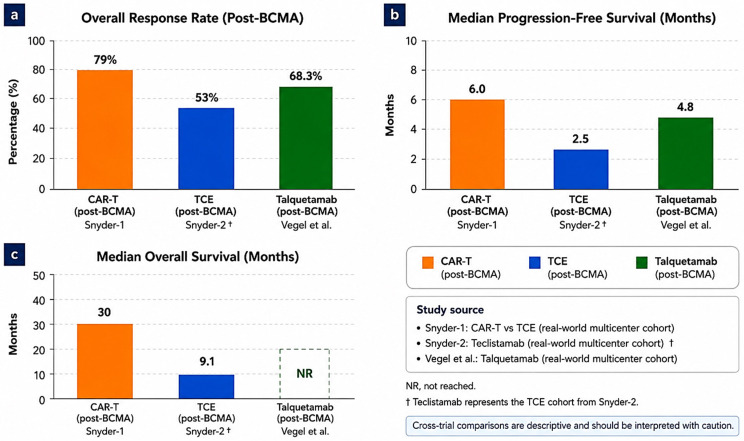
Cross-trial summary of response and survival outcomes following BCMA-directed therapy. Bar plots illustrate overall response rate (ORR; (**a**)), median progression-free survival (PFS; (**b**)), and median overall survival (OS; (**c**)) across CAR-T therapy, BCMA T-cell engagers (TCEs), and talquetamab in the post-BCMA setting. Data are derived from independent multicenter real-world cohorts [18,19,20]. Cross-trial comparisons are descriptive and should be interpreted with caution given differences in patient populations, prior therapies, and study designs.

In the univariate analysis, teclistamab administered ≤ 6 months after BDT was associated with inferior outcomes (PFS HR 2.5 [95% CI, 1.29–4.84]; OS HR 2.91 [95% CI, 1.43–5.91]). Additional adverse prognostic factors included 1q amplification (hazard ratio [HR] 1.62, 95% CI 1.06–2.47), and double-refractory disease (HR 2.15, 95% CI 1.06–4.46) (defined as refractoriness to both proteasome inhibitors and immunomodulatory agents). Baseline characteristics and outcomes of BDT-exposed versus BDT-naïve patients treated with teclistamab in Snyder-2 are summarized in Table 2. Furthermore, comparative distributions of ORR, PFS, and OS across BCMA-directed CAR-T versus TCE therapy in Snyder-1 and teclistamab cohorts in Snyder-2 are illustrated in Figure 1.

### 3.3. Vegel et al.’s Study: Talquetamab in Heavily Pretreated Patients with MM, Including BDT-Refractory [20]

The talquetamab cohort included 68 patients, of whom 63 had had prior exposure to BDT at seven US academic centers. The median age of the whole population was 69 years (38–86), and the Revised International Staging System (R-ISS) stage III disease was 23.5%. The most common prior BDT class was TCE (77.8%) followed by CAR-T (68.3%), and 9.5% received ADC. The population was highly refractory (triple-class, 88.2%; penta-drug, 70.6%). Among patients previously exposed to BDT, 82.5% were refractory.

Among patients previously exposed to BDT, the ORR to talquetamab was 68.3%, including ≥VGPR in 47.6% and complete response or better in 22.2%. Patients with prior exposure to BCMA bispecific TCEs had a significantly lower ORR than did those with no prior TCE exposure (59.2% vs. 100%; *p* < 0.01). In addition, receipt of BDT within 6 months before talquetamab initiation or as the immediately preceding therapy was associated with lower ORR (56.8% vs. 84.6%; *p* = 0.02 and 48% vs. 80.6%; *p* < 0.01, respectively) and shorter PFS. The median PFS survival in the BDT cohort was 4.8 months, whereas the median OS was not reached at a median follow-up of 4.6 months.

**Figure 2:**
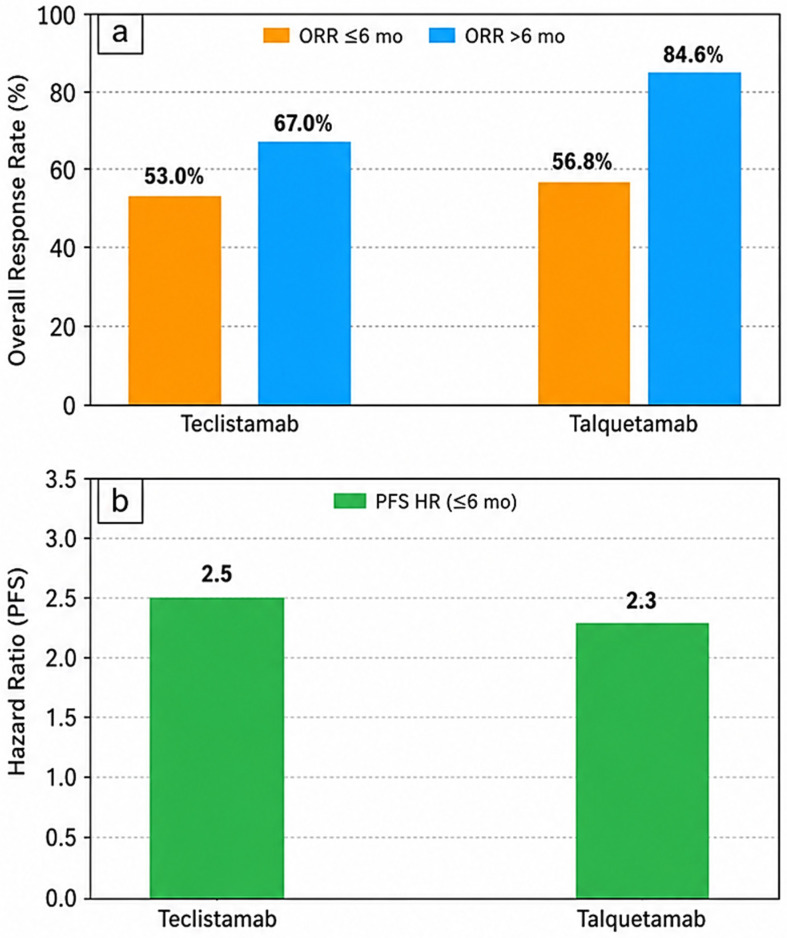
Impact of treatment-free interval on overall response rate and progression free survival by therapy. (**a**) Shorter intervals (≤6 months) after BCMA therapy were associated with lower ORR for both teclistamab (53.0% vs. 67.0%) and talquetamab (56.8% vs. 84.6%). (**b**) Shorter intervals (≤6 months) were associated with higher risk of progression for both teclistamab (hazard ratio [HR] 2.5; 95% CI 1.29–4.84) and talquetamab (HR 2.3; *p* = 0.03). These findings suggest an association between longer treatment-free intervals (≥6 months) and improved outcomes.

Adverse events were consistent with clinical trial data and included cytokine release syndrome (63.2%), immune effector cell-associated neurotoxicity syndrome (11.8%), dysgeusia (77.9%), and skin-related events (51.5%). Grade 3/4 hematologic toxicities included leukopenia (11.2%), thrombocytopenia (23.6%), and anemia (15.3%). Baseline characteristics and outcomes of patients treated with talquetamab after BDT in this study are summarized in Table 3. The impact of the treatment-free interval on ORR and PFS across the teclistamab and talquetamab cohorts is illustrated in Figure 2.

## 4. Discussion

The introduction of BDT has revolutionized the management of RRMM, offering meaningful and durable responses in heavily pretreated patients. As these agents are increasingly incorporated earlier in the therapeutic course, relapse after BDT exposure is emerging as a significant clinical challenge. Understanding how to effectively sequence subsequent therapies after BDT failure has therefore become a key unmet need. While prior studies have largely evaluated individual agents or therapeutic classes in isolation, the present analysis offers a focused synthesis of selected three multicenter, real-world studies, across US academic centers in collaboration with the USMIRC network, to provide insights and hypothesis-generation into post-BDT outcomes, highlighting comparative, real-world synthesis across multiple post-BCMA treatment approaches, including BCMA-directed CAR-T therapy, BCMA bispecific antibodies, and non-BCMA–targeted therapies such as talquetamab.

Importantly, our findings extend beyond individual efficacy estimates by identifying consistent patterns across therapeutic platforms. This approach enables a comparative perspective across multiple immunotherapeutic strategies, including BCMA-directed CAR-T therapy, BCMA bispecific antibodies, and non-BCMA–targeted therapies such as talquetamab. Collectively, this focused real-world synthesis provides pragmatic insights into post-BDT sequencing in the absence of prospective randomized data, while underscoring the need for larger, systematic, and controlled studies to validate these observations.

A comparative overview of BCMA-directed CAR-T therapies, BCMA TCEs, and non-BCMA antigen-switch strategies in BDT-exposed patients with RRMM is shown in Table 4.

### 4.1. Comparative Efficacy of CAR-T and TCEs After BDT

In this analysis, BCMA-directed CAR-T therapy was associated with higher response rates, deeper responses, and longer OS compared with BCMA TCEs when used after BDT exposure. These findings are consistent with prior reports of durable responses with ide-cel and cilta-cel in heavily pretreated populations [3,13]. Importantly, patients able to proceed to CAR-T therapy often exhibit more favorable disease biology and slower disease kinetics, allowing sufficient time for leukapheresis and manufacturing. In contrast, patients with rapidly progressive disease are frequently treated with off-the-shelf TCEs because they need immediate disease control [23]. This biologic and kinetic selection likely contributes, at least in part, to the superior outcomes observed with CAR-T therapy and should be considered when interpreting cross-modality comparisons. Despite these advantages, real-world barriers—including manufacturing time, toxicity monitoring requirements, hospitalization needs, caregiver support, and eligibility constraints—continue to limit CAR-T accessibility. Notably, real-world data indicate that BCMA-directed CAR-T therapy retains comparable efficacy in patients with baseline renal impairment—despite higher rates of immune effector cell-associated neurotoxicity syndrome and infections—supporting the feasibility of cellular therapy in carefully selected patients with renal dysfunction [27]. Furthermore, retrospective analyses in penta-refractory multiple myeloma demonstrate that exposure to BCMA-directed therapies is associated with significantly improved overall survival compared with non-BCMA approaches (median OS 17 vs. 6 months), underscoring the critical role of BCMA-targeted strategies in this heavily pretreated population [28]. Accordingly, TCEs remain an essential therapeutic option, particularly for patients who require rapid disease control or are ineligible for cellular therapy.

### 4.2. Impact of Treatment Interval and Resistance Mechanisms

Both the teclistamab and talquetamab cohorts exhibited inferior outcomes when subsequent immunotherapy was administered within 6 months of prior BDT [29]. This finding supports the hypothesis that closely sequenced immune-redirecting therapies may be limited by cumulative immune dysfunction, including T-cell exhaustion and impaired effector fitness [30]. In the teclistamab cohort, patients previously exposed to BDT experienced lower response rates and shorter PFS, likely reflecting a combination of adverse disease biology and therapy-induced immune impairment.

T-cell exhaustion is increasingly recognized as a key resistance mechanism after T-cell–redirecting therapies, particularly with continuous bispecific antibody exposure, where chronic antigen stimulation promotes upregulation of inhibitory receptors and progressive loss of cytotoxic function [31]. This immune dysfunction may diminish responsiveness to subsequent immunotherapies. Consistent with this model, patients with longer treatment-free intervals exhibited better outcomes, underscoring the potential importance of immune recovery between sequential immune-based treatments (Figure 2).

Importantly, the association between shorter treatment-free intervals (≤6 months) and inferior outcomes is likely multifactorial and not solely attributable to immune dysfunction. Early relapse following BDT may also reflect inherently aggressive disease biology, driven by clonal evolution and selection of therapy-resistant subclones [32,33]. Multiple myeloma is characterized by significant spatial and temporal heterogeneity, and therapeutic pressure can promote expansion of resistant clones with adverse genomic features, including high-risk cytogenetic abnormalities [34]. These biological processes contribute to a more proliferative and treatment-refractory phenotype, which independently predicts poorer clinical outcomes [33].

From a clinical perspective, patients requiring early retreatment often exhibit features of aggressive disease, including high tumor burden, extramedullary involvement, and heavily pretreated or refractory disease states. These factors are well-established predictors of inferior survival and may confound the observed association between shorter treatment intervals and reduced efficacy of subsequent therapies [33]. Therefore, the inferior outcomes observed with closely sequenced immunotherapies likely reflect a composite effect of both impaired immune fitness and unfavorable underlying disease biology.

However, prolonged treatment-free intervals are not feasible for many patients with aggressive RRMM. Therefore, alternative strategies may be required, including finite-duration or response-adapted bispecific therapy, sequencing of agents with nonoverlapping targets, and earlier incorporation of antigen-switch approaches [35,36]. Ongoing investigation into optimized scheduling and novel targets will be critical to balancing disease control with preservation of immune fitness.

### 4.3. Teclistamab Use After BDT

Teclistamab retained meaningful activity in BDT-exposed patients, although outcomes were inferior to those of BDT-naïve populations. These real-world findings are consistent with MajesTEC-1 and other datasets, which revealed diminished efficacy after prior BDT use. In MajesTEC-1, previously BDT-exposed patients achieved an ORR of 52.5% and median PFS of 4.5 months [10,24]. Additional real-world analyses have shown similarly shortened PFS in this population [37,38]. Notably, multicenter real-world data demonstrate that teclistamab maintains comparable response rates and toxicity profiles in patients with baseline renal impairment—albeit with increased transfusion requirements—supporting its use in medically complex RRMM populations with appropriate monitoring [39].

The reduced efficacy of teclistamab after prior BDT exposure likely reflects both tumor-intrinsic and immune-mediated resistance mechanisms, including BCMA modulation or loss, potential BCMA genomic alterations, and cumulative T-cell dysfunction from prior immune-redirecting therapies [40,41]. Longer intervals between BDT may permit partial restoration of T-cell fitness and, in some cases, recovery of antigen expression [31]. Although routine assessment of BCMA density or mutation status is not yet standard, expanding molecular profiling may enable more biologically informed sequencing strategies in the future [40].

Emerging data also suggest that fixed duration teclistamab after achievement of deep response may be feasible in appropriately selected patients, with early survival outcomes comparable to continuous therapy. This approach may help mitigate cumulative T-cell exhaustion and infection risk associated with prolonged exposure. However, prospective studies are needed to define optimal patient selection criteria, immune recovery biomarkers, and appropriate discontinuation timing [42].

### 4.4. Talquetamab as a Non-BDT Alternative

Talquetamab, a GPRC5D-targeting bispecific antibody, represents an important antigen-switch strategy for patients relapsing after BDT. In this real-world cohort, talquetamab demonstrated robust activity in heavily pretreated and BDT-exposed patients, consistent with prior clinical trial and real-world reports [25,26]. Notably, outcomes were inferior when talquetamab was administered shortly after BDT discontinuation, further supporting the concept that recent immune-redirecting therapy may induce transient T-cell dysfunction that reduces subsequent treatment responsiveness.

### 4.5. Belantamab-Based Triplets in Early Relapse

In the DREAMM-7 and DREAMM-8 phase III trials, belantamab mafodotin–based triplet regimens were evaluated in the second-line setting for relapsed/refractory multiple myeloma. In DREAMM-7, the combination of belantamab mafodotin, bortezomib, and dexamethasone (BVd) demonstrated a clinically meaningful improvement in progression-free survival (PFS) compared with daratumumab, bortezomib, and dexamethasone (DVd). Similarly, in DREAMM-8, belantamab mafodotin, pomalidomide, and dexamethasone (BPd) significantly improved PFS compared with pomalidomide, bortezomib, and dexamethasone (PVd) (hazard ratio [HR], 0.52; 95% confidence interval [CI], 0.37–0.73; *p* < 0.001), with an early trend toward improved overall survival (HR, 0.77; 95% CI, 0.53–1.11) [43,44].

Despite these favorable outcomes, established second-line standards—particularly carfilzomib- or lenalidomide-based triplet regimens—remain widely preferred in clinical practice due to their well-characterized survival benefit, more predictable safety profiles, and the absence of treatment-related ocular toxicity requiring specialized monitoring.

### 4.6. Sequencing Considerations and Clinical Implications

The optimal sequencing of therapies after BDT remains a dynamic and evolving challenge in RRMM [29]. Our integrated analysis supports several key principles: (1) Therapy type matters; CAR-T therapy provides superior depth and durability of response after BDT exposure and should be prioritized when clinically feasible. (2) Timing is critical; treatment-free intervals exceeding 6 months between sequential BDTs are associated with improved PFS and OS. (3) Antigen switching enhances durability; non-BCMA targets, such as GPRC5D (talquetamab) or FcRH5, represent effective antigen-switch strategies, offering meaningful responses in BDT-refractory disease.

**Figure 3:**
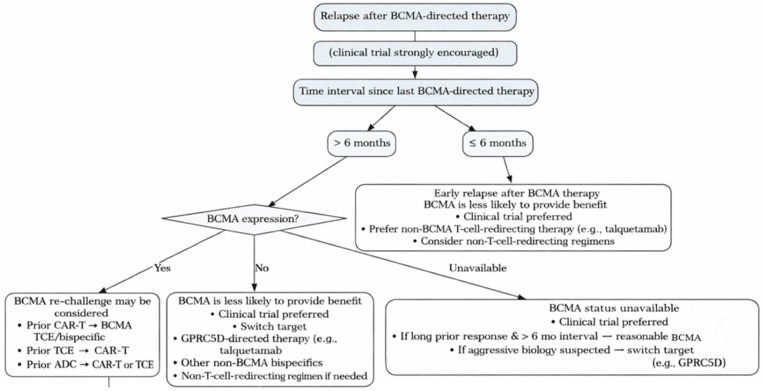
Post-BCMA therapy suggested sequencing algorithm. Proposed decision framework for patients with relapsed/refractory multiple myeloma after BCMA-directed therapy. Management is guided primarily by the interval since prior BCMA exposure. Early relapse (≤6 months) favors switching to non-BCMA strategies or clinical trial enrollment, whereas later relapse (>6 months) may allow consideration of BCMA re-challenge, depending on BCMA expression and prior therapy.

These findings highlight the importance of applying personalized therapeutic sequencing, informed by prior exposure, disease kinetics, and immune recovery, to optimize long-term outcomes for patients with RRMM after BDT. A proposed algorithm that summarizes post-BDT therapeutic sequencing after MM relapse is illustrated in Figure 3 and should be interpreted as a conceptual framework based on the current analysis.

This study has inherent limitations, including its retrospective, nonrandomized design with potential selection bias, heterogeneity in treatment selection and response assessment across centers, and reliance on unadjusted exploratory analyses. Additionally, the included studies differed in eligibility criteria, patient populations, prior treatment exposures, and analytic methodologies, which limits direct comparability and reinforces the descriptive, hypothesis-generating nature of cross-study comparisons. Differences in disease burden, number of prior lines of therapy, and sequencing strategies may introduce confounding and affect external validity. Furthermore, modest subgroup sample sizes reduced statistical power and precluded definitive causal inference regarding optimal therapeutic sequencing after BDT. These limitations highlight the need for prospective, controlled studies to better define optimal sequencing strategies in the post-BDT setting.

## 5. Conclusions

In this multicenter real-world analysis, BCMA-directed CAR-T therapy was associated with the most favorable outcomes in selected patients with prior BDT exposure, and treatment timing and antigen selection emerged as critical determinants of subsequent therapeutic success. Short treatment intervals between immune-redirecting therapies were consistently associated with inferior outcomes, whereas antigen-switch strategies such as talquetamab provide an effective option for patients with BDT-refractory disease.

Prospective studies are urgently needed to refine sequencing algorithms, define optimal treatment-free intervals, and incorporate immunologic and genomic biomarkers to guide personalized therapy selection. Such efforts will be essential to maximize the long-term benefit of next-generation immunotherapies in MM.

## Figures and Tables

**Table 1:** Baseline characteristics and outcomes of BCMA CAR-T versus BCMA TCEs after BCMA-directed therapy [18].

Characteristic/Outcome	BCMA CAR-T (*n* = 42)	BCMA TCE (*n* = 53)	*p* Value
Type of therapy received			
Ide-cel	32	–	–
Cilta-cel	10	–	–
Teclistamab	–	52	–
Elranatamab	–	1	–
Baseline characteristics			
Median age, years (range)	66 (42–83)	66 (42–83)	NR
R-ISS stage III, %	26	28	NR
High-risk cytogenetics (per IMWG criteria), %	43	57	NR
EMD, %	43	42	NR
Triple-refractory disease, %	86	92	NR
Prior BCMA-directed therapy exposure, %			
ADC	64	23	–
TCE	29	–	–
CAR-T	–	75	–
Outcomes			
ORR, %	79	51	<0.001
≥VGPR, %	64	47	<0.001
Median PFS, months (95% CI)	6 (5–14)	2 (1–8)	0.06
Median OS, months (95% CI)	30 (30–NR)	12 (7–NR)	0.008

Abbreviations: ADC, antibody–drug conjugate; BCMA, B-cell maturation antigen; BDT, BCMA-directed therapy; CAR-T, chimeric antigen receptor T-cell therapy; CI, confidence interval; EMD, extramedullary disease; ide-cel, idecabtagene vicleucel; cilta-cel, ciltacabtagene autoleucel; IMWG, International Myeloma Working Group; NR, not reported; ORR, overall response rate; OS, overall survival; PFS, progression-free survival; R-ISS, Revised International Staging System; TCE, T-cell engager; VGPR, very good partial response. Notes: Baseline characteristics are presented as reported in the original study without additional statistical comparison. *p*-values are reported for outcome comparisons where available. “–“ indicates not applicable.

**Table 2:** Baseline characteristics and outcomes of BDT-exposed versus BDT-naïve patients treated with teclistamab [19].

Characteristic/Outcome	BDT-Exposed (*n* = 60)	BDT-Naïve (*n* = 104)	*p* Value
Baseline characteristics			
Median age, years (range)	66 (37–84)	69 (46–87)	0.092
R-ISS stage III, %	28	28	–
Autologous SCT, %	75	59	0.035
Number of prior lines of therapy, mean (range)	7 (6–9)	4 (4–6)	<0.001
Triple-class refractory disease, %	88	73	0.022
Prior ASCT, %	75	59	0.035
Prior BCMA-directed therapy exposure, %			
CAR-T	68	–	–
ADC	37	–	–
TCE	8.3	–	–
Outcomes			
ORR, %	53	68	0.02
Median PFS, months (95% CI)	2.5 (1.4–8.9)	9.7 (4.5–NR)	0.01
Median OS, months (95% CI)	9.1 (6.1–NR)	NR	0.08
Exploratory analyses (BDT-exposed cohort only)			
OS HR (95% CI)	2.91 (1.43–5.91)	–	–
Amplification 1q HR (95% CI)	1.63 (1.06–2.47)	–	–
Double refractory HR (95% CI)	2.15 (1.06–4.46)	–	–

Abbreviations: ASCT, autologous stem cell transplant; ADC, antibody–drug conjugate; BCMA, B-cell maturation antigen; BDT, BCMA-directed therapy; CAR-T, chimeric antigen receptor T-cell therapy; CI, confidence interval; HR, hazard ratio; NR, not reached; ORR, overall response rate; OS, overall survival; PFS, progression-free survival; R-ISS, Revised International Staging System; TCE, T-cell engager. Notes: *p*-values are reported for comparisons between BDT-exposed and BDT-naïve groups where applicable. “–” indicates not applicable. Exploratory hazard ratios are reported for the BDT-exposed cohort only, as described in the original study.

**Table 3:** Baseline characteristics and outcomes of patients treated with talquetamab after BCMA-directed therapy [20].

Characteristic/Outcome	Talquetamab Cohort (*n* = 63)	*p* Value
Baseline characteristics		
Median age, years (range)	69 (38–86)	–
R-ISS stage III, %	23.5	–
Number of prior lines of therapy, median (range)	7 (4–17)	–
Prior BCMA-directed therapy exposure, %		
CAR-T	68.3	–
TCE	77.8	–
ADC	9.5	–
Refractory to prior BCMA-directed therapy, %	82.5	–
Outcomes		
ORR, %	68.3	–
≥VGPR, %	47.6	–
CR, %	22.2	–
Subgroup and exploratory analyses		
ORR by prior TCE exposure	59.2% vs. 100%	<0.01
Impact of ≤6 months interval from prior BDT		
ORR	56.8% vs. 84.6%	0.02
PFS HR	2.32	0.03
OS HR	4.82	0.04

Abbreviations: ADC, antibody–drug conjugate; BCMA, B-cell maturation antigen; BDT, BCMA-directed therapy; CAR-T, chimeric antigen receptor T-cell therapy; CR, complete response; HR, hazard ratio; ORR, overall response rate; OS, overall survival; PFS, progression-free survival; R-ISS, Revised International Staging System; TCE, T-cell engager; VGPR, very good partial response. Notes: As this represents a single-cohort study, *p*-values are reported only for subgroup and exploratory comparisons as provided in the original study. “–” indicates not applicable.

**Table 4:** Key studies evaluating the sequencing of BCMA- and non-BCMA-directed therapies in patients with RRMM.

Study	Therapy Type(s)	Population	Prior BCMA Exposure	Key Outcomes	Key Findings/ Implications
A. CAR-T–Based Therapy (Post-BCMA)					
Snyder et al., 2025 [18]	CAR-T (ide-cel, cilta-cel) vs. TCE (teclistamab, elranatamab)	95 RRMM pts post-BDT	100%	ORR 79% vs. 51%; PFS 6 mo vs. 2 mo; OS 30 mo vs. 12 mo	CAR-T achieved superior depth and durability; preferred where feasible.
CARTITUDE-2 (Cohort C) [23]	Cilta-cel (BCMA-CAR-T)	Post-BCMA (ADC or TCE)	100%	ORR 60%; MRD-neg 7/10 at 10^−5^; mDOR 11 mo	Demonstrates CAR-T efficacy even after prior BCMA therapy; supports adequate interval before reuse.
B. BCMA-Directed TCEs					
Snyder et al., 2024 [19]	Teclistamab (BCMA × CD3 bispecific)	164 RRMM pts (60 prior BDT)	37%	ORR 53% vs. 68%; PFS 2.5 vs. 9.7 mo; OS 9.1 mo vs. NR	Efficacy reduced post-BCMA; ≤6-mo interval predicts poorer PFS/OS; ≥6 mo interval recommended.
MajesTEC-1 (Cohort C) [24]	Teclistamab (BCMA × CD3 bispecific)	40 RRMM pts with prior BCMA (ADC: *n* = 29; CAR-T: *n* = 15; both: *n* = 4)	100%	ORR 52.5%; ≥VGPR 47.5%; CR 30%; mPFS 4.5 mo; mOS 15.5 mo	Confirms reduced activity post-BCMA; >6-mo interval improves outcomes.
MagnetisMM-1 (Phase I) [11]	Elranatamab (BCMA × CD3 bispecific)	88 RRMM pts	~15–20%	ORR 63.6%; CR 38.2%; PFS 11.8 mo; OS 21.2 mo	Durable responses: activity retained in some post-BCMA cases.
MagnetisMM-3 (Cohort B) [5]	Elranatamab (BCMA × CD3 bispecific)	64 RRMM pts post-BCMA (ADC/CAR-T)	100%	ORR ~46% (interim)	Moderate activity post-BCMA; supports reuse after interval.
C. Non-BCMA/Antigen-Switch Therapies					
Vegel et al., 2025 [20]	Talquetamab (GPRC5D-TCE)	63 RRMM pts with prior exposure to BCMA-directed therapy	82.5% refractory	ORR 68.3%; ≥VGPR 47.6%; CR 22.2%	Antigen switching effective; efficacy lower if ≤6 mo interval.
Chari et al., 2022 [25]	Talquetamab (GPRC5D × CD3 bispecific)	232 RRMM pts (102 IV, 130 SC)	~30%	ORR 70% (405 µg weekly) and 64% (800 µg q2wk); ≥VGPR ~55–57%; CR ~29–30%; median DOR: 10.2 mo (weekly)/7.8 mo (q2wk)	Strong non-BCMA activity; supports antigen-switch strategy; manageable CRS (77–80%).
Jakubowiak et al., 2025 [26]	Talquetamab (GPRC5D × CD3 bispecific)	185 heavily pretreated RRMM pts (incl. BCMA-refractory)	100%	ORR 71% (overall); 68% in BCMA-exposed; mPFS 4.8 mo; ≥VGPR 47%; CR 22%	Confirms talquetamab efficacy post-BCMA; ≤6 mo interval reduces response; reinforces antigen-switch and timing principles.

Abbreviations: ADC, antibody–drug conjugate; BCMA, B-cell maturation antigen; BDT, BCMA-directed therapy; CAR-T, chimeric antigen receptor T-cell therapy; CR, complete response; CRS, cytokine release syndrome; DOR, duration of response; GPRC5D, G protein-coupled receptor, family C, group 5, member D; IV, intravenous; MRD, minimal residual disease; NR, not reached; ORR, overall response rate; OS, overall survival; PFS, progression-free survival; RRMM, relapsed/refractory multiple myeloma; SC, subcutaneous; TCE, T-cell engager; VGPR, very good partial response.

## Data Availability

As our manuscript is based on a descriptive synthesis of three previously conducted multicenter studies. We did not generate new data nor have access to individual participant-level datasets, and all analyses were performed using data already reported in the original publications. Accordingly, no new datasets were created or analyzed that could be shared.
